# Gallium-Based Liquid Metals: From Properties to Applications

**DOI:** 10.3390/nano16080471

**Published:** 2026-04-16

**Authors:** Zhonggui Li, Xinyi Han, Xiaoyu Guo, Le Ma, Jialin Sun, Yaokuan Wen, Yao Guo

**Affiliations:** Centre for Quantum Physics, Key Laboratory of Advanced Optoelectronic Quantum Architecture and Measurement, School of Physics, Beijing Institute of Technology, Beijing 100081, China

**Keywords:** liquid metals, gallium, biocompatibility, catalysis, sensors

## Abstract

Gallium-based liquid metals have garnered significant attention due to their distinct combination of metallic and liquid behavior at room temperature. This review systematically examines the fundamental properties and advanced multifunctional applications of this class of materials. Key characteristics such as low melting point, excellent fluidity, high electrical and thermal conductivity, and biocompatibility are first highlighted. Subsequently, progress in four major application areas is discussed. In sensing, these materials enable the fabrication of highly compliant and responsive devices capable of monitoring strain, temperature, and electromagnetic fields. Within biomedical engineering, their inherent low toxicity and biocompatibility underpin advances in biosensing platforms, precision drug delivery, and engineered tissue scaffolds. For energy-related applications, they are utilized in batteries and high-efficiency thermoelectric systems for converting heat into electricity. In catalysis, their dynamic and tunable interfaces facilitate efficient carbon dioxide conversion and selective thermocatalytic reactions. This review summarizes current advances in the application of gallium-based liquid metals and provides critical perspectives on future developments and opportunities in this technology.

## 1. Introduction

In advanced functional material systems, liquid metal (LM) materials that combine metallic properties with fluid dynamics have become a frontier focus in inter-disciplinary research. Their exceptional physicochemical characteristics transcend the application limitations of traditional solid materials. With the increasing demand for multifunctionality, environmental adaptability, and miniaturization in modern industry, LMs have application potential in pivotal fields such as electronics [1,2,3,4], biomedicine [5,6,7,8,9], energy storage [10,11,12,13], and catalysis [14,15,16,17]. This drives innovation in materials science and engineering technology.

Gallium-based LMs, as prominent representatives of the LM family, are primarily composed of gallium. Their properties are regulated through alloying, specifically via the incorporation of indium or tin. These alloys exhibit distinct properties, including a low melting point [18,19], high thermal and electrical conductivity [20,21], biocompatibility [22,23,24], and shape deformability [25,26,27,28,29,30,31,32]. Compared with traditional LMs like mercury, gallium-based LMs are non-toxic and environmentally friendly [21,24]. The paramount value of such materials resides in their concurrent unification of two intrinsically antithetical attributes: superior electrical and thermal conductivity characteristic of metals, and rheological deformability inherent to liquids. The metallic component ensures efficient electron transport, signal transduction, and energy transfer, whereas the fluidic component confers arbitrary shape adaptability, interfacial conformability, dynamic reconfigurability, and autonomous self-healing. This dualistic nature underpins tunable interfacial phenomena, adaptive responsiveness, and multifunctional integration capacity, thereby establishing a robust foundation for innovative, cross-disciplinary applications.

**Figure 1:**
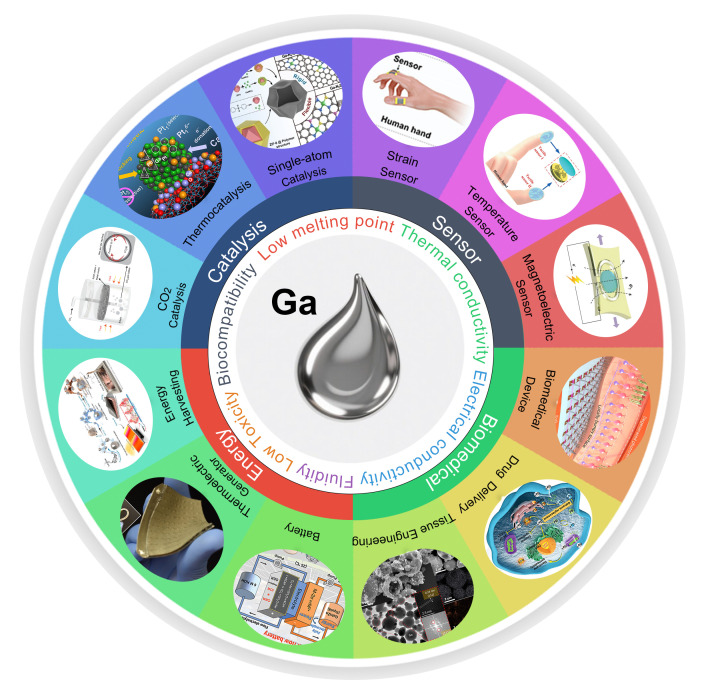
Schematic highlighting the recent advances in LM applications [75,76,77,78,79,80,81,82,12,83,84,85]. Copyright 2025, Advanced Functional Materials. Copyright 2021, International Journal of Smart and Nano Materials. Copyright 2020, Advanced Functional Materials. Copyright 2024, Nature Nanotechnology. Copyright 2023, Advanced Materials. Copyright 2025, Advanced Functional Materials. Copyright 2024, Advanced Energy Materials. Copyright 2020, ACS Applied Materials & Interfaces. Copyright 2022 ACS Nano. Copyright 2022, Energy & Environmental Science. Copyright 2021, Angew. Chem. Int. Ed. Engl. Copyright 2023, Angew. Chem. Int. Ed. Engl.

Research on gallium-based LMs has transitioned from fundamental exploration to practical implementation [33,34,35,36,37,38,39,40], with applications spanning intelligent sensing, biomedicine, energy utilization, and catalysis. Benefiting from the synergistic effect of high electrical/thermal conductivity and deformability, the intrinsic properties of LMs can be directly translated into device functions. High conductivity provides the physical basis for sensing, energy, and catalysis. Meanwhile, fluidity and deformability enable devices to adapt to flexible substrates, dynamic tissues, complex microstructures, and wearable systems. This combination achieves integration and service unavailable with traditional solid materials. For sensing applications, their high mechanical–electrical response sensitivity facilitates the accurate detection of physical and chemical signals [1,41,42,43,44,45,46,47,48,49]. In terms of biomedicine, favorable biocompatibility endows them with unique advantages in drug delivery [50,51,52,53,54,55], tumor therapy [56,57,58,59], and bioimaging [5,6,8,23,60,61]. Regarding energy utilization, superior electrical conductivity opens up new avenues for developing high-efficiency energy storage devices [13,62,63,64,65] and thermal management systems [66,67,68,69,70]. As for catalysis, their tunable surface active sites, abundant interfacial active centers, and dynamic interfacial behaviors hold the potential to surmount the performance limitations of traditional catalytic materials [15,71,72,73,74]. As shown in Figure 1, this review goes beyond focusing on a single field such as flexible electronics, adopting a broader materials science perspective to present cutting-edge advances in four application areas: sensing, biomedicine, energy, and catalysis. It systematically maps the key properties of gallium-based LMs, namely their low melting point, fluidity, electrical conductivity, and biocompatibility, to their cross-disciplinary applications. Thus, it integrates fundamental properties with diverse applications, highlighting the multifunctionality and inter-disciplinary value of gallium-based LM materials.

## 2. Properties of Gallium-Based Liquid Metals

The distinctive physicochemical properties of gallium-based LMs constitute the foundational basis for their advanced applications across multiple fields. At room temperature, these materials synergistically combine fluidity [86,87], high electrical and thermal conductivity [20,88], favorable biocompatibility [22], and low toxicity [34]. This combination enables them to meet the demands of deformation control and functional integration in complex environments.

### 2.1. Low Melting Point

Low melting point stands as a prominent characteristic of gallium-based LMs, laying an essential prerequisite for their processing, performance regulation, and applications across multiple fields. Gallium, a post-transition metal, has a melting point of merely 29.76~29.8 °C [89,90]. Its distinctive low-temperature-melting feature stems from dual structural and bonding traits: on one hand, the outer electronic configuration of gallium atoms gives rise to a unique bonding state where strong covalent bonds coexist with weak metallic bonds [89]; on the other hand, diatomic Ga_2_ molecules exist in elemental gallium, and the melting process only requires overcoming weak intermolecular van der Waals forces, enabling the transition from solid to liquid at relatively low temperatures [91].

By means of alloying regulation with other post-transition metals such as indium and tin, the melting point performance of gallium-based LMs can be further optimized, broadening their applicability at low temperatures. X-ray absorption spectroscopy reveals an expansion of atomic bond lengths in Ga-In and Ga-Sn alloys compared to pure gallium, which is associated with the disruption of the layered gallium network and contributes to the observed melting point depression [89,92,93]. Among typical eutectic alloys, eutectic gallium–indium (EGaIn, Ga75~75.5%, In24.5~25%, mass fraction) has a melting point as low as 15.7 °C, and Galinstan (Ga68~68.5%, In21.5~22%, Sn10%, mass fraction) boasts an even lower melting point of −19 °C. In addition, alloy series such as GaIn, GaZn, GaAl, GaSn, and AlGaIn all exhibit melting points lower than that of elemental gallium [89,94,95]. Gallium-based LMs also possess a notable supercooling effect, retaining a liquid form even when the temperature drops well below their melting points, further expanding its application scenarios at low temperatures [36].

### 2.2. Fluidity

One of the important properties of LMs are their fluidity. The primary expression of this trait lies in their ability to flow, undergo deformation and reconfigure themselves under external forces [96]. Regarding fundamental physical characteristics, pure gallium boasts a viscosity comparable to that of water, thus maintaining good fluidity. It has a melting point of 29.8 °C and a supercooling capacity reaching 70 °C, allowing it to stay liquid and retain flowability across a specific temperature interval beneath its melting point [97,98]. At room temperature EGaIn exhibits a viscosity of 1.99 mPa·s, roughly double that of water, and a density around 6.3 times higher than that of water [99]. Upon exposure to ambient atmosphere, a self-limiting oxide skin with thickness of 0.7–3 nm forms within seconds on the surface, transforming the LM into a non-Newtonian fluid with a yield stress. Under low stress, the oxide skin behaves as an elastic solid, maintaining non-spherical configurations. Once the critical stress is exceeded, the skin ruptures and the low-viscosity metallic core flows instantaneously. The oxide skin contributes to mechanical properties two orders of magnitude higher than the bulk viscous resistance, rendering the macroscopic rheological behavior almost entirely governed by the surface oxide rather than the bulk metal.

The fluid nature of gallium-based LMs, mediated by their surface oxide, underlies their electrochemically addressable interfacial properties and dynamic responsivity. The surface of gallium-based LMs spontaneously forms a nanoscale oxide layer under ambient conditions, which acts as an intrinsic surfactant to drastically reduce interfacial tension from ~500 mJ/m^2^ to near zero under mild electrochemical or oxidative stimuli [100]. This surface oxide layer exhibits reversible yielding, fracture, and self-recovery behavior under mechanical stress, electric field, or pH changes, enabling dynamic modulation of interfacial tension, wettability, adhesion, and contact impedance [101,102,103]. Furthermore, the liquid nature allows continuous atomic rearrangement and fast structural reconfiguration, enabling adaptive conformal contact with complex surfaces, flexible substrates, and biological tissues. The synergy between bulk fluidity, electrochemically tunable surface oxide, and reversible interfacial tension variation constitutes the fundamental physical and rheological mechanism underlying the tunable interface and dynamic responsiveness of gallium-based LMs [104,105,106].

### 2.3. Electrical Conductivity

Gallium-based LMs are materials rich in free electrons that retain excellent metallic electrical conductivity even in the liquid state, emerging as highly promising prominent materials in the field of flexible electronics. Both pure gallium and common gallium-based alloys exhibit outstanding electrical performance: the electrical conductivity of an EGaIn alloy reaches 3.4 × 10^6^ S/m, while that of a gallium–indium–tin ternary alloy attains 3.46 × 10^6^ S/m [97,107,108]. Although their conductivity is an order of magnitude lower than that of copper, it is considerably higher than that of typical ionic liquids, demonstrating remarkable conductive advantages [109].

In terms of the conductive mechanism, the abundance of free electrons within gallium-based LMs serves as the fundamental basis for their high conductivity. Even in the long-range disordered atomic structure of the liquid state, the transport of free electrons remains stable [110,111]. Furthermore, their electrical resistance varies linearly with temperature, laying the groundwork for the controllability of their electrical conductivity [112,113]. It is worth noting that when gallium-based LMs are exposed to oxygen, a metal oxide layer rapidly forms on their surface, which increases interfacial resistance and thus impairs electrical conductivity [101,109,114]. This oxidation-induced conductivity loss is fully reversible. It is thermodynamically unstable in both strong acidic and strong alkaline environments. Under such conditions, the surface oxide layer can react with hydrogen ions or hydroxide ions to generate water-soluble gallium complexes. These reactions gradually decompose and completely dissolve the oxide film that increases interfacial resistance. The original highly conductive LM surface is thereby fully exposed [101,115,116]. This effective oxide removal mechanism reliably restores the intrinsic electrical conductivity of gallium-based LMs.

Gallium-based LMs combine room-temperature fluidity with excellent metallic conductivity. They fundamentally solve the key problem of simultaneous mechanical and electrical failure in traditional conductive materials under dynamic deformation [34]. Thin films of solid metals such as copper and silver tend to fracture brittlely under strain. In contrast, gallium-based LMs can achieve an ultra-large strain. They remain fatigue-free after millions of loading cycles [117,118]. They completely eliminate mechanical fatigue and microcrack growth caused by accumulated lattice distortion in rigid materials. More importantly, their fluid nature provides the system with unique electrical self-healing ability. When microcracks appear in the solid conductive layer of composite structures, LMs can bridge the broken paths instantly through controlled release. Electrical healing can be triggered under a tiny bending strain. These materials also withstand extreme tensile deformation. Their performance is significantly better than traditional solid conductors or pure LM particle composites [119]. In addition, microstructures such as liquid–solid double layers and porous frameworks can be applied. The materials maintain metallic conductivity and stable strain-insensitive resistance output. They offer an ideal mechanoelectrical integrated material foundation for wearable, implantable and highly reliable flexible electronic systems.

### 2.4. Thermal Conductivity

The thermal conductivity of gallium-based LMs stems from efficient heat transfer by free electrons within the metallic structure [120]. These materials exhibit thermal conductivity significantly superior to that of non-metallic heat transfer media such as air and water: the thermal conductivity of pure gallium reaches 30.5 W·m^−1^·K^−1^, while those of EGaIn and Galinstan are 26.43 W·m^−1^·K^−1^ and 25.41 W·m^−1^·K^−1^, respectively [121,122]. This surpasses the thermal conductivity of water by more than 40 times and exceeds that of most inorganic and organic phase change materials by two orders of magnitude, enabling rapid heat transfer and efficient heat absorption–release processes [17,123,124].

The impact of the oxide layer formed on the surface of gallium-based LMs on their thermal conductivity is far less pronounced than that on solid metals. In contrast to solid metals, whose thermal conductivity typically decreases by 8 to 12 times after oxidation, the thermodynamically stable β-Ga_2_O_3_ oxide layer on gallium-based LMs has a thermal conductivity ranging from 11 to 27 W·m^−1^·K^−1^, which is comparable to that of the base material and thus does not significantly impair heat transfer efficiency [124,125]. Furthermore, these materials possess high volumetric heat capacity and low saturated vapor pressure. Coupled with their wide operating temperature range spanning from 10 °C to over 2000 °C and flexibly tunable phase transition temperature, they demonstrate remarkable application potential in scenarios such as battery thermal management and microdevice cooling [101,126,127].

### 2.5. Low Toxicity and Biocompatibility

The low toxicity and biocompatibility of gallium-based LMs serve as a major driver for their extensive attention in the biomedical field, and current research has yielded a relatively clear understanding of these properties [108,128]. Compared with typical toxic LMs such as mercury, the low toxicity of gallium-based LMs first stems from their distinctive physicochemical characteristics: their vapor pressure is extremely low at room temperature [129]. Elemental gallium reaches a 1 Pa vapor pressure only at 1037 °C, while the EGaIn alloy remains below 1.33 × 10^−10^ Pa at 300 °C. By contrast, mercury reaches 1 Pa at merely 42 °C. Consequently, gallium-based metals pose minimal risk via gaseous diffusion. Additionally, their negligible water solubility further mitigates toxic potential. Various forms of gallium-based LMs and their derivatives exhibit low toxicity in terms of cytotoxicity, hepatotoxicity, hemotoxicity, and histotoxicity [130,131]. Although non-essential, gallium exhibits favorable biocompatibility at low doses. Its inability to penetrate erythrocytes precludes interference with oxygen transport. In physiological environments, gallium undergoes degradation or dissolution, releasing Ga (III) ions via redox or oxidative enzymatic reactions, with subsequent efficient renal and fecal excretion.

However, potential adverse biological effects of gallium-based LMs have also been documented. Human exposure to gallium halides can lead to acute poisoning accompanied by dermatitis, tachycardia, dyspnea and irreversible cardiomyopathy [132]. Inhaled gallium oxide particles have been shown to induce persistent pulmonary inflammation, impaired lung clearance, alveolar proteinosis and progressive fibrosis in rats [133]. Eutectic gallium–indium LMs can release cytotoxic gallium and indium ions under mechanical agitation and cause concentration- and time-dependent cell damage in several human cell lines [134].

## 3. Sensors Built with LM

Gallium-based LMs integrate flexible mechanical properties, high electrical conductivity and favorable environmental responsiveness, acting as a highly applicable functional material in the realm of flexible sensors [2,3,135,136]. They exhibit promising application performance in the sensing fields of strain [119,137,138,139], temperature [140,141], magnetoelectricity [77,142,143], pressure [144,145] and humidity [146,147,148,149], effectively compensating for the partial application limitations of conventional materials. This section reviews the operating principles and research advances of these representative sensors.

Gallium-based LMs overcome the challenge of synergizing electrical and mechanical properties in sensing materials. They enhance the performance of stretchable sensors and related devices. Structured bulk metals rely on geometric strain engineering for extensibility. Even the optimized fractal structure can achieve a high degree of stretching strain. Their essence remains pseudo-extensibility. Beyond the 5% pre-strain threshold, they are prone to cracking and delamination [150]. Metal brittleness also limits fatigue life. Intrinsically stretchable materials face a trade-off between conductivity and stretchability. Conductive polymers exhibit conductivity three to five orders of magnitude lower than metals. Silver nanowire networks suffer from poor cyclic stability [34]. Composite hydrogels can achieve high stretchability [151], yet their conductivity remains two to three orders of magnitude lower than that of LMs. Their preparation is also complex. Gallium-based LMs circumvent these trade-offs through intrinsic fluidity. They can achieve ultra-large tensile strain and millions of fatigue-free cycles. Their conductivity reaches 3.4 × 10^6^ S/m, comparable to solid metals [99]. Microchannel design enables highly linear responses. The oxide skin confers configuration adaptability. They also possess electrical self-healing capability under tiny strain. This function is unattainable by other materials. LM sensors achieve synergistic optimization in conductivity, stretchability, linearity, fatigue life, and self-healing [30,100]. They play important functional roles in various sensing applications.

### 3.1. Strain Sensor

**Figure 2:**
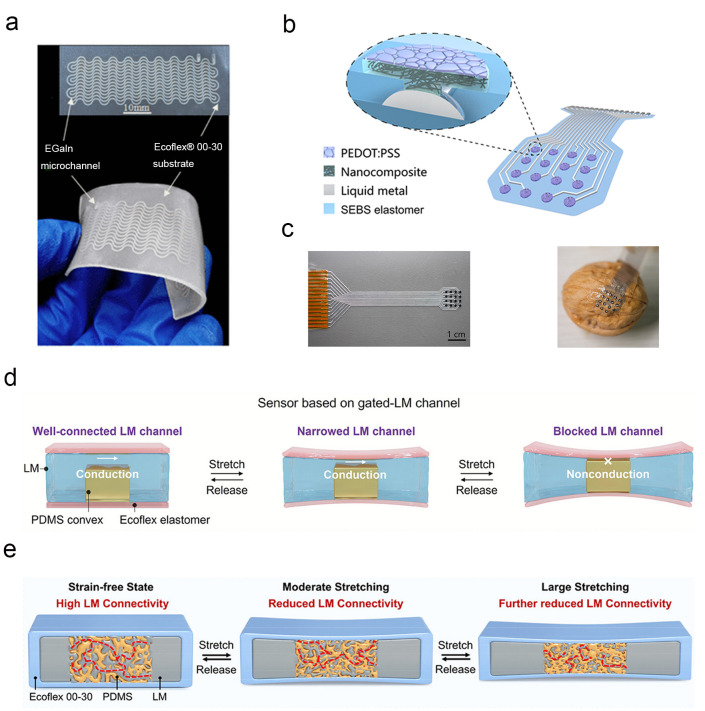
(**a**) Optical image of the enhanced LM-based microfluidic strain sensor [152]. Copyright 2020, American Chemical Society. (**b**) Schematic illustration of the device architecture involving elastomeric encapsulation, LM interconnects, nanocomposite electrodes, and microcracked conductive polymer interface. (**c**) Optical image of an as-prepared electronic patch connected to a flexible polyimide connector [153]. Copyright 2022, Science Advances. (**d**) Designed structure and principle of the strain sensor [154]. Copyright 2024, Advanced Functional Materials. (**e**) Structure changes and strain-dependent LM connectivity of the proposed sensor during stretching. In the strain-free state, the LM-filled foam exhibits high connectivity. Moderate stretching reduces LM connectivity, while large stretching further disrupts the pathways, leading to increased resistance. The red dashed lines indicate the LM conduction paths, whose connectivity changes with applied strain [75]. Copyright 2025, Advanced Functional Materials.

Microfluidic strain sensors based on conventional materials are often limited by hysteresis caused by elastomer viscoelasticity and interfacial molecular forces, which severely impairs measurement accuracy and reliability. Wave-patterned structures can regulate the deformation behavior of elastomers via spatial topological constraints, effectively suppressing energy dissipation induced by viscoelasticity. By embedding eutectic gallium–indium LM into wave-shaped elastomeric microchannels of specific dimensions, Chen et al. significantly reduced the sensor’s hysteresis and endowed it with excellent stretch tolerance (Figure 2a). Further optimization by extending the microchannel length substantially improved sensitivity and microstrain detection capability, enabling applications in human activity monitoring and robot joint motion tracking [152]. Implantable sensors require the formation of a conformal interface with dynamic biological tissues, demanding both ultra-high stretchability and physiological stability along with low interfacial impedance. The intrinsic liquid nature of LMs enables unlimited deformation, and their excellent biocompatibility perfectly meets the core requirements of implantable scenarios. Wang et al. constructed an implantable device with an all-flexible material system, using LM as interconnect components [153]. As shown in Figure 2b,c, the device achieved ultra-high tensile strain and outstanding durability against repetitive deformations, while its metal-level conductivity ensured minimal signal loss. Demonstrating long-term stability under physiological conditions, the device successfully completed electrophysiological mapping of rapidly beating hearts, providing important technical support for the diagnosis of cardiovascular diseases.

LM-based strain sensors often suffer from intrinsic nonlinearity in resistance response and insufficient sensitivity, while traditional improvement methods involving rigid particle embedding tend to cause interfacial mismatch and compromise stability. In Figure 2d, Yao et al. proposed a foam-gating mechanism, embedding soft elastomeric foam infused with LM into LM channels and fully encapsulating the assembly. The foam acts as a strain-responsive unit to regulate electronic transport pathways: in the strain-free state, the LM maintains high connectivity and low resistance; under tensile deformation, foam compression reduces connectivity, ultimately achieving a highly linear response over a wide strain range with minimal signal drift during long-term use [154]. The sensitivity enhancement of LM sensors is often restricted by resistance changes arising solely from dimensional effects, while the introduction of rigid components undermines long-term stability and flexibility. Based on the Poisson effect, heterogeneous elastomers undergo differential deformation under stress: soft materials are prone to cross-sectional contraction, whereas the geometric shape of rigid materials remains essentially unchanged. Yao et al. introduced stiffer PDMS protrusions into soft elastomeric channels filled with LM [75]. These protrusions function as strain-modulated gates, with the design illustrated in Figure 2e. During stretching or pressing, the soft channels undergo significant cross-sectional contraction, leading to a substantial elevation in sensor sensitivity. The all-soft design ensures the device’s stability under prolonged and repetitive deformations, enabling multimodal sensing of stretching, pressing, and bending. It has been successfully applied to the detection of subtle human movements and reliable monitoring of soft gripper grasping actions.

### 3.2. Thermal–Mechanical Dual-Parameter Sensor

**Figure 3:**
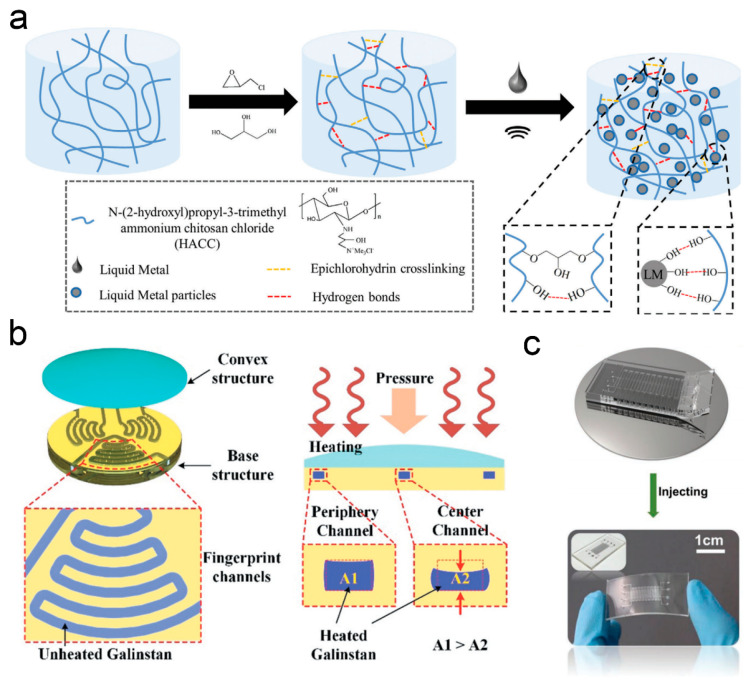
(**a**) Schematic illustration of the preparing process of the chitosan quaternary ammonium salt and LM hydrogel [155]. Copyright 2022, Macromolecular Rapid Communications. (**b**) Two-layered structure of the sensor and fingerprint-patterned microfluidic channels with embedded Galinstan LM; temperature and contact force sensing principles [76]. Copyright 2021, International Journal of Smart and Nano Materials. (**c**) Optical image of the stretchable dual-parameter sensor [156]. Copyright 2022, Advanced Materials Technologies.

A challenge in developing materials for thermal–mechanical dual-parameter sensing lies in achieving high biocompatibility, multimodal sensing precision, and seamless integration with biological tissues or soft robots. Such capabilities are difficult to realize with conventional rigid electronic materials. Biomass polymers can form stable hydrogel matrices through physicochemical crosslinking. The combination of their natural biocompatibility and the flexible conductive properties of LMs enable the synergistic enhancement of sensing functions and biological adaptability to address the need for skin-conformable, multifunctional interfaces that can operate without delamination or signal interference. In Figure 3a, Wang et al. constructed a crosslinked chitosan quaternary ammonium salt–LM composite hydrogel by compounding renewable chitosan quaternary ammonium salt with gallium-based LMs [141,155]. Benefiting from the synergistic effect between the two components, the material integrates temperature–stress bimodal sensing, antibacterial activity, and self-healing performance. It can be directly attached to the skin for monitoring physiological movements and realizing human–computer interaction control.

Digital light processing 3D printing technology can precisely regulate the molding process of photosensitive resins. Dual-material printing allows the construction of microchannel structures with heterogeneous hardness, providing a stable carrier for the encapsulation and sensing of LMs. The room-temperature fluidity of LMs enables them to seamlessly fill microchannels, and independent identification of different signals can be achieved with dedicated circuit design. Wang’s team adopted a dual-material digital light processing printing process to prepare sensor substrates containing microchannels, injecting gallium-based LMs as the sensing core. As depicted in Figure 3b, simultaneous capture of temperature and force signals was realized through circuit decoupling [76]. The fabricated sensor possesses both flexibility and durability, suitable for bimodal detection in scenarios such as robotic grasping.

To overcome the challenge of signal crosstalk inherent in monolithic bimodal sensors, where deformation and temperature responses often interfere with one another, multilayer stacked structures can achieve physical isolation of different sensing units. Capacitive sensing units are deformation-sensitive, while resistive sensing units respond to temperature; their combination enables simultaneous bimodal detection through signal conversion. Gallium-based LM microelectrodes exhibit excellent signal transmission efficiency. Zhang et al. designed a layered stacked sensor structure, independently arranging capacitive deformation units and resistive temperature units and embedding gallium-based LM microelectrodes for signal conversion [156] (see Figure 3c). Structural optimization ensures the detection accuracy of bimodal parameters, making it applicable for real-time monitoring of various physiological activities and complex working conditions.

### 3.3. Magnetoelectric Sensor

**Figure 4:**
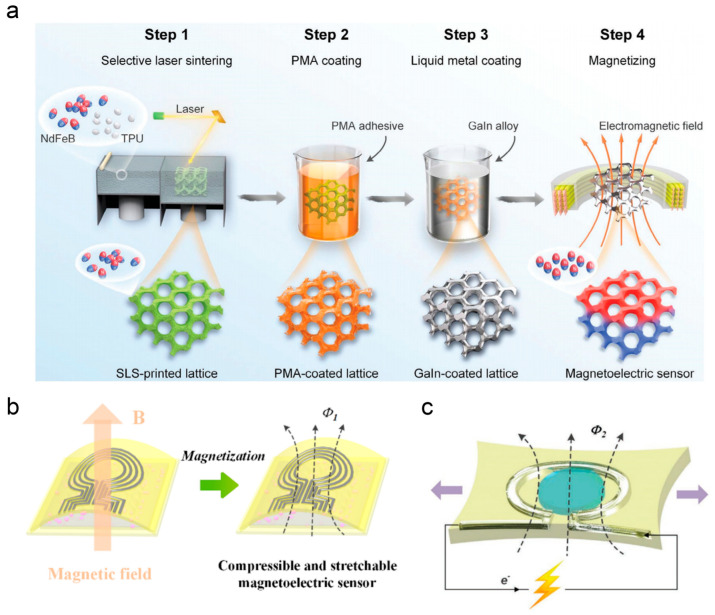
(**a**) Schematic diagram illustrating the process of fabricating flexible magnetoelectric sensors using selective laser sintering combined with subsequent LM 3D transfer printing [143]. Copyright 2024, Advanced Materials. (**b**) A compressible and stretchable magnetoelectric sensor with an arch air-layer gap was prepared via integrating the magnetic and electrical parts, followed by a magnetization process for electrical tests [157]. Copyright 2021, ACS Applied Materials & Interfaces. (**c**) The secondary encapsulation of Ecoflex mixture was undertaken before a stretchable magnetoelectric film was prepared [77]. Copyright 2021, ACS Advanced Functional Materials.

Magnetoelectric sensors play an indispensable role in flexible electronics and healthcare monitoring. Electromagnetic induction underpins the self-powered operation of magnetoelectric sensors, enabling the conversion of mechanical energy into electrical energy through external force-induced changes in magnetic flux. Additive manufacturing technology allows for the structural customization of sensors, while LMs with their metallic conductivity and liquid-state deformability provide a material foundation for addressing the challenges of efficient integration between conductive and magnetic components while ensuring flexibility. Wu et al. developed a hybrid process combining selective laser sintering and 3D transfer printing: they first fabricated magnetic lattice structures via selective laser sintering, then used 3D transfer printing to form a conformal conductive network of LM on the lattice surface, as illustrated in Figure 4a [143]. Meanwhile, interface modification resolved the adhesion issues between LM and the polymer substrate, and a relevant model was established for the accurate calculation of magnetic flux. This design achieved the simultaneous enhancement of structural flexibility and magnetoelectric integration of the sensor, enabling responses to multi-directional external forces.

In respiratory monitoring, a pressing demand exists for self-powered sensors that combine high sensitivity with multi-directional response capability, as traditional piezoelectric or triboelectric sensors often suffer from insufficient sensitivity and rely on external power sources, making it difficult to accurately capture complex air pressure variations during breathing. As shown in Figure 4b, Zhang et al. designed an arch-shaped air-gap compressible and stretchable magnetoelectric sensor, using LM and magnetic powders as functional materials [157]. Leveraging the low Young’s modulus of these materials, the sensor achieves the dual sensing capabilities of compression and stretching. The introduction of an air layer precisely regulates the distance of electromagnetic interaction, enabling rapid response and excellent stability for non-invasive respiratory monitoring scenarios.

Wearable electronic devices require sensors to have large-strain adaptability and self-powered capability, which depends on the effective combination of the deformability of conductive materials and energy conversion principles. In electromagnetic induction, changes in electromagnetic interaction caused by the relative motion between components serve as the basis for mechanoelectrical conversion in stretching scenarios. Zhang et al. developed gallium-based LM stretchable magnetoelectric films, replacing conventional rigid conductive materials with LM [77] (see Figure 4c). During cyclic stretching–releasing processes, the relative motion between components regulates electromagnetic interaction to achieve self-powered mechanoelectrical conversion. Combined with programmed patterning and structural optimization, the electrical performance of the sensor can be tailored on demand, meeting the requirements of large-strain wearable applications. The performance of flexible energy harvesters has been constrained by limitations in energy conversion efficiency and impedance matching inherent to traditional piezoelectric materials, which rely on surface charge accumulation and often face issues related to piezoelectric coefficients and impedance mismatch. In contrast, magnetoelectric composites generate charges through the relative motion between magnetic and conductive components, offering new pathways for improving conversion efficiency and optimizing impedance. Chen et al. fabricated a soft magnetoelectric energy harvester based on electromagnetic induction, free of piezoelectric materials [142]. Its internal impedance exhibits excellent compatibility with commercial electronic devices, and the structural design achieves ultra-high energy conversion efficiency with simplified fabrication and reduced costs. Gallium-based LMs provide fundamental support for the flexibilization and high-performance advancement of magnetoelectric sensors.

## 4. Biomedical Engineering

Compared with traditional rigid metal electrodes such as copper and silver–silver chloride, gallium-based LMs feature excellent fluidity, ductility and self-healing ability due to their liquid nature. They form conformal contact with dynamic biological tissues. This significantly reduces motion artifacts and interfacial impedance. Their impedance is significantly lower than that of Ag/AgCl electrodes [158]. The release of gallium ions provides intrinsic antibacterial, antitumor and osteogenic activities. This breaks the limitation of traditional materials that only have a single conductive function [1,159]. Gallium-based LMs enable the construction of integrated diagnosis and treatment platforms. Their low melting point supports flexible manufacturing processes such as injection molding and 3D printing. These properties facilitate the development of novel medical systems including wearable physiological signal monitoring devices, implantable neural interfaces and biodegradable transient electronics. These comprehensive characteristics make gallium-based LMs a key candidate material to overcome the rigidity bottleneck of existing bioelectronic devices and achieve long-term stable human–machine interfaces. The biomedical field imposes stringent requirements on the biocompatibility and physicochemical property tunability of functional materials. LMs featuring flexibility and low cytotoxicity are well-suited to biomedical application scenarios [5,7,144,145,146,147]. This section elaborates on their latest applications in three research areas: biomedical devices, drug delivery, and tissue engineering.

### 4.1. Biomedical Device

**Figure 5:**
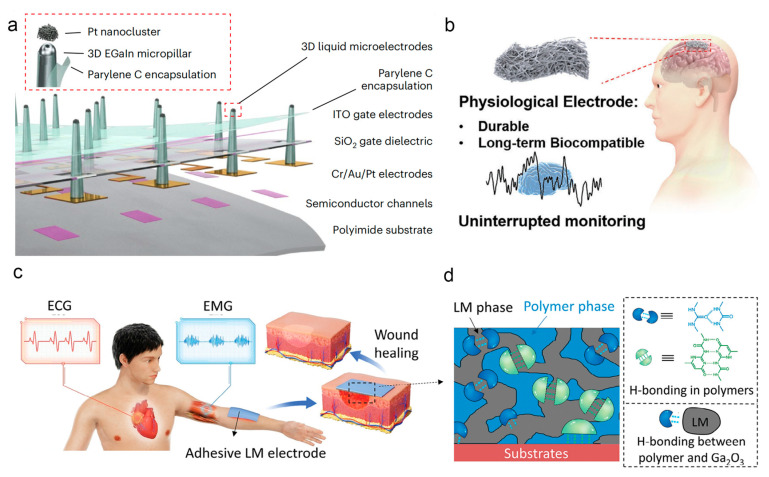
(**a**) Schematic of the layouts of the artificial retina based on the integration of photosensitive transistors with 3D LM microelectrodes [78]. Copyright 2024, Nature Nanotechnology. (**b**) Schematic illustration of a durable and long-term biocompatible physiological electrode application [160]. Copyright 2025, Advanced Materials. (**c**) Schematic illustrations of LM electrodes with a broad range of tunable adhesion for epidermal biopotential monitoring and accelerating wound healing [162]. Copyright 2024, Advanced Functional Materials. (**d**) Schematic structure of the LM/UPy8-PEI electrode [162]. Copyright 2024, Advanced Functional Materials.

A major challenge in retinal implants lies in the mechanical mismatch between rigid electrodes and soft neural tissues, which often causes tissue damage, while excessive electrode-to-target-cell distance impairs stimulation selectivity. Gallium-based LMs, with their low modulus, can conform to soft retinal tissue, and three-dimensional structural design reduces the distance between electrodes and ganglion cells. In Figure 5a, Chung developed an ultrathin and flexible artificial retina employing epiretinal implantation to minimize invasiveness; tip modification of electrodes enhances charge injection efficiency, and unsupervised machine learning aids in analyzing neural signals [78]. In vivo experiments demonstrated spatiotemporal mapping of neural responses to localized illumination, offering a viable approach for vision restoration. Long-term application of implantable physiological electrodes relies on favorable biocompatibility and structural stability, with permeability being critical for unobstructed tissue metabolism and enhanced biocompatibility. Despite excellent electrical conductivity and stability, gallium-based LMs tend to aggregate and detach in porous substrates due to poor wettability. Zhou addressed this issue by pre-modifying porous substrates with silver nanoparticles to regulate LM wettability, fabricating a permeable and durable liquid-metal fiber mat electrode [160]. As shown in Figure 5b, this electrode enables stable acquisition of physiological signals, mitigating adverse tissue reactions associated with long-term implantation of conventional devices and providing a reliable option for prolonged physiological monitoring.

Peripheral nerve interfaces face the challenge of adapting to the flexibility and stretchability of biological tissues, as conventional rigid or semi-flexible electrodes fail to match the mechanical behavior of neural tissues, often leading to unstable signal transmission or tissue damage. The fluidity of LMs allows them to withstand repeated stretching while maintaining electrical continuity. Tang developed a gallium-based LM fluidic cuff electrode, which maintains stable transmission of sciatic nerve signals and delivers effective stimulation during long-term in vivo implantation in freely moving animals [161]. This device overcomes the mechanical mismatch limitations of conventional electrodes, laying a foundation for the advancement of artificial peripheral nerves. Epidermal bioelectronics demand balanced electrical stability, tissue adhesion, and biocompatibility, where conventional LM electrodes lack integration of multiple performance attributes. As shown in Figure 5c,d, dynamic interactions between ureidopyrimidinone-modified polymers and LMs enable simultaneous regulation of adhesion strength and formation of conductive networks. Cao developed LM electrodes with tunable adhesion. These electrodes establish stable connections with rigid components while serving as comfortable wound-interfaced electrodes, expanding the application scenarios of LM electrodes in multifunctional biomedical devices [162]. Gallium-based LM devices have found applications across diverse biomedical scenarios, with notable advances in structural design and performance optimization.

### 4.2. Drug Delivery

The advancement of precision in smart drug delivery systems is central to enhancing therapeutic efficacy and reducing side effects. Gallium-based LMs, possessing low toxicity, excellent biocompatibility, and tunable physicochemical properties, have emerged as promising candidates to overcome the limitations of traditional carriers, exhibiting remarkable advantages in targeted drug delivery and controlled release.

**Figure 6:**
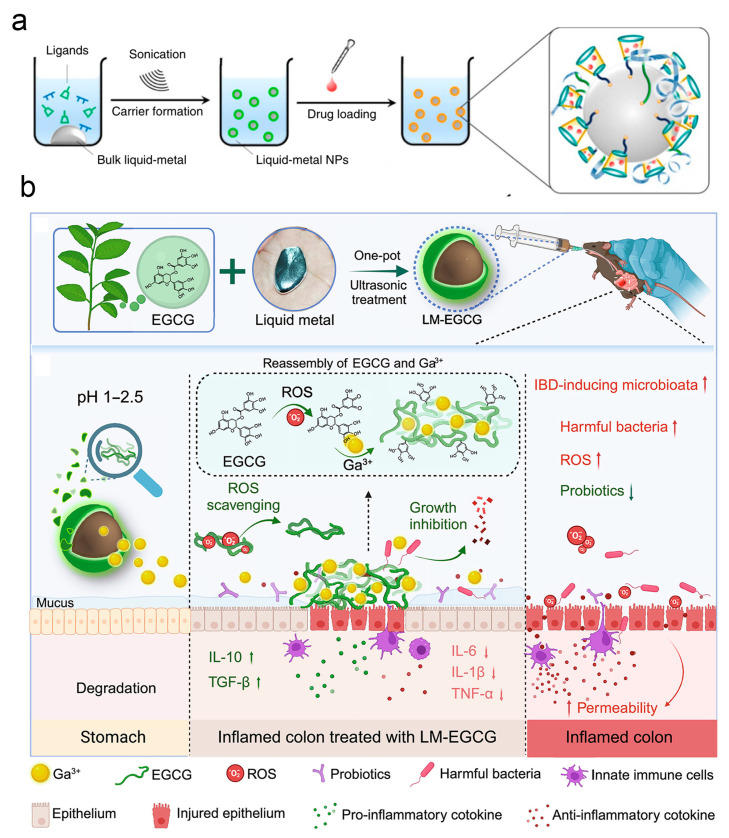
(**a**) Preparation route of LM-NP/Dox-L [163]. Copyright 2015 Nature Communications. (**b**) LM–EGCG was successfully constructed by one-pot ultrasonic treatment and orally administered for IBD therapy. Also shown is the process of pH-mediated degradation of LM–EGCG in the stomach and reassembly of EGCG with Ga^3+^, thereby modulating the dysregulated microbiome, intestinal barrier, and immune responses for alleviating inflammation diseases [164]. Copyright 2024, Science Advances.

Achieving precise drug release within the tumor microenvironment while overcoming multidrug resistance remains a persistent limitation of conventional carriers. Stimuli-responsive drug delivery systems achieve precision drug release by recognizing specific signals in the tumor microenvironment, among which the mildly acidic condition serves as a widely used endogenous trigger. Gallium-based LMs can undergo fusion and degradation in acidic environments, providing a favorable material foundation for constructing intelligent delivery carriers. Lu et al. designed a core–shell structured carrier with a EGaIn alloy as the inner phase [163] (see Figure 6a). Through an ultrasound-mediated ligand assembly strategy, thiolated cyclodextrin was used as the drug-loading matrix and hyaluronic acid as the tumor-targeting ligand. After cellular endocytosis, the carrier undergoes fusion and degradation in acidic endosomes to release drugs; meanwhile, the degradation products can synergistically reverse tumor drug resistance, offering a novel approach for the development of low-toxicity tumor theranostic agents.

Clinical treatment of inflammatory bowel diseases has long faced numerous challenges. Orally administered drugs are prone to rapid clearance, resulting in low bioavailability. Additionally, traditional drugs have limited efficacy in regulating gut microbiota, making it difficult to restore intestinal microecological balance and thus affecting treatment prognosis. As shown in Figure 6b, Liu integrated the similarity in physicochemical properties between gallium ions and iron ions with the adhesive and coordination capabilities of polyphenolic compounds, constructing an oral LM formulation encapsulated by epigallocatechin gallate (EGCG) [164]. Following targeted adhesion to inflamed tissues, the formulation directly scavenges reactive oxygen and nitrogen species and enhances local accumulation by forming EGCG–gallium complexes, thereby regulating microbiota balance and protecting intestinal barrier function to achieve precision treatment of inflammatory bowel diseases.

### 4.3. Tissue Engineering

**Figure 7:**
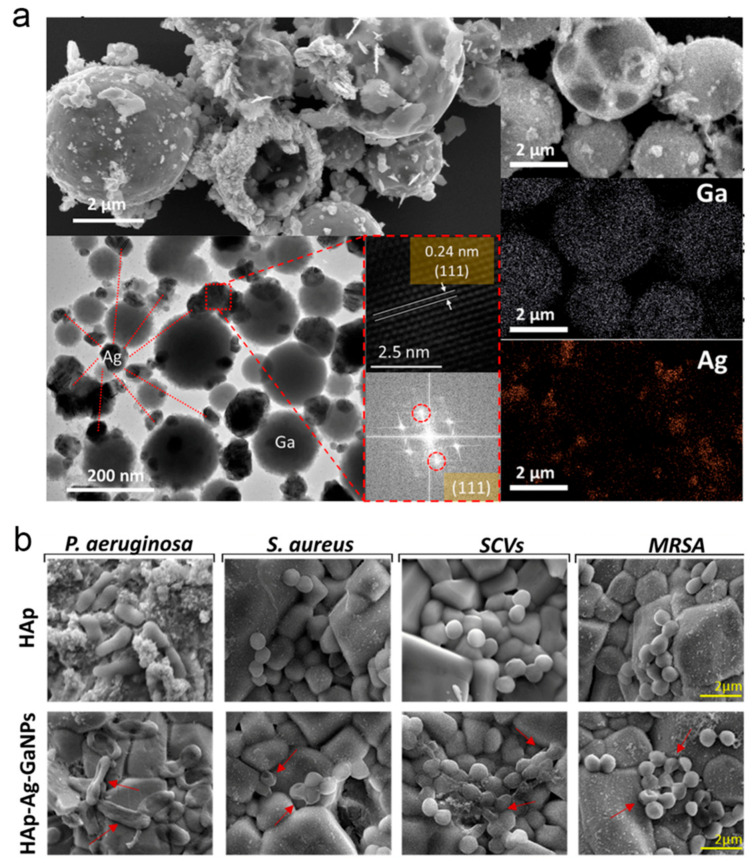
(**a**) Morphological analysis using scanning electron microscopy and transmission electron microscopy shows spherical nanoparticles of HAp-Ag-GaNPs, with high-resolution transmission electron microscopy confirming the presence of AgNPs and GaNPs through lattice fringes (0.24 nm, corresponding to the (111) plane). Elemental mapping highlights the homogeneous distribution of Ag and Ga within the nanoparticle. The red box highlights the Ag (111) plane [80]. Copyright 2025, Advanced Functional Materials. (**b**) SEM images showing morphological alterations in bacterial morphology are observed on HAp-Ag-GaNPs surfaces compared to HAp. Red arrows point to bacterial cell damage and lysis [80]. Copyright 2025, Advanced Functional Materials.

Implant-associated infections in orthopedics tend to result in implant failure, as conventional hydroxyapatite (HAp) coatings lack potent antibacterial activity. As shown in Figure 7a, Nguyen et al. designed HAp composite coatings modified with silver–gallium LM nanoparticles, building on the antibacterial activity of silver ions and the iron-mimetic properties of gallium ions [80]. This coating disrupts vital bacterial metabolic processes through multiple mechanisms while preserving the osteogenic activity of HAp. It has been demonstrated to reduce bacterial colonization and enhance tissue integration, offering a novel strategy for integrated infection control and osteogenesis in orthopedic implants (see Figure 7b). During bone repair, the stiffness of defect sites undergoes dynamic changes as tissue regenerates, and static scaffolds struggle to meet this physiological requirement. Gallium-based LMs possess both hydrogel-like plasticity and metal-like mechanical strength and can achieve external regulation through the incorporation of magnetic particles. Li et al. developed magnetic LM scaffolds and porous magnetic LM scaffolds by combining magnetic particles with Galinstan for the first time [165]. These scaffolds achieve dynamically tunable stiffness via magnetic field regulation, and their favorable biocompatibility promotes osteogenic differentiation of stem cells, providing a new platform for simulating dynamic mechanical microenvironments. Multiscale structures in natural tissues are essential for functional performance, yet conventional manufacturing technologies struggle to simultaneously construct macroscopic and microscopic features in soft biomaterials. Gallium-based LMs have a melting point close to cell culture temperatures and their surface oxide layer can be regulated under mild conditions, endowing them with advantages as a sacrificial template. Sundaram et al. proposed engineered sacrificial capillary pumps for evacuation molding technology, using gallium-based LMs as a template [166]. Leveraging capillary forces for complete template removal, this technology successfully constructs multiscale branched structures in natural hydrogels, offering a fresh approach to the in vitro fabrication of complex tissue architectures.

## 5. Energy

The development and application of special functional materials in the energy sector constitute an essential driver for advancing energy storage and the high-efficiency utilization of energy, where LMs exhibit considerable application potential [10,11,12,13,62,167,168]. The application value of gallium-based LMs in the energy field comes from the combination of their liquid nature and wide-temperature stability. In battery energy storage, liquid electrodes avoid dendrite problems through alloying reaction mechanisms. They also show self-healing ability. These features improve the cycle life of batteries. Their medium-temperature working performance and heat recovery design help achieve high system energy efficiency. In the field of thermoelectric power generation, their excellent wettability and electromagnetic pump driving technology solve interface matching problems of rigid modules. They support waste-heat recovery in wearable devices and complex industrial scenarios. In energy recovery applications, their wide liquid temperature range and no-moving-part design allow direct recovery of medium- and high-temperature waste heat. This also improves the reliability of the whole system. Compared with traditional materials, gallium-based LMs have core advantages. Their flexible and self-healing properties break through the mechanical limits of solid materials. Their wide-temperature adaptability meets the application requirements of various scenarios. Their biological safety supports the needs of sustainable development [98,109]. This section focuses on two directions of their applications in the energy field: acting as electrode or interface materials for batteries to elevate energy storage density and operational safety, and fabricating flexible thermoelectric generators (TEGs) based on the Seebeck effect to realize efficient recovery and utilization of heat.

### 5.1. Battery

**Figure 8:**
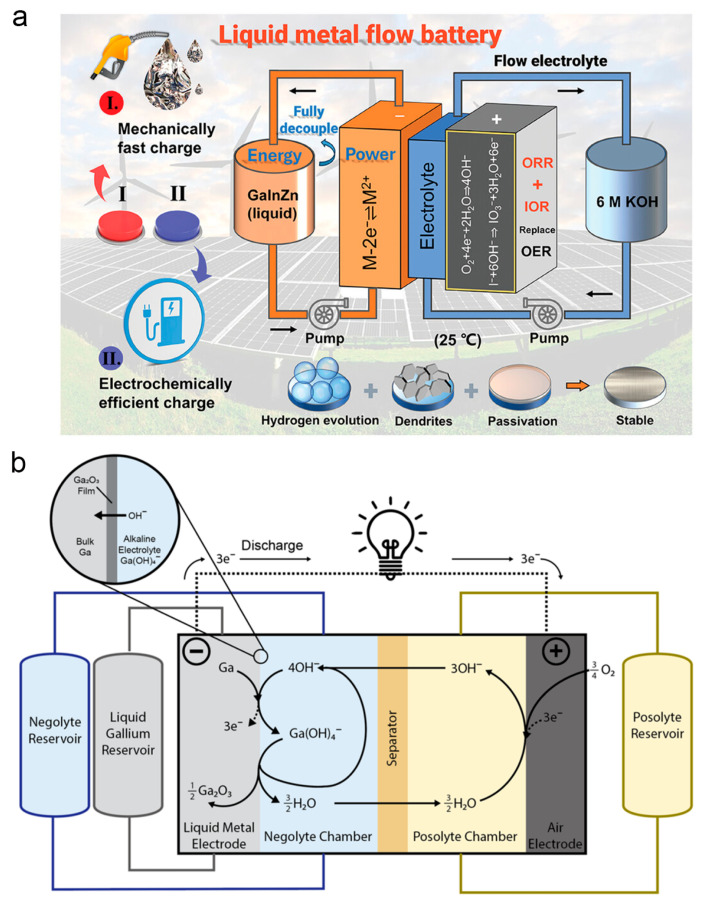
(**a**) A high-energy-density room-temperature LM-based flow battery supporting rapid mechanical charging as well as conventional electrochemical charging [81]. Copyright 2024, Advanced Energy Materials. (**b**). Schematic of a LM-air battery utilizing liquid gallium as it goes through a redox cycle mediated by soluble gallium oxide [169]. Copyright 2024, ACS Applied Energy Materials.

The performance enhancement of flow batteries hinges on the high reversibility of electrodes, fast reaction kinetics and suppression of side reactions. Conventional flow batteries face persistent challenges such as dendrite growth and hydrogen evolution, which compromise cycling stability and safety, while the decoupling of energy and power remains structurally constrained. The liquid nature of gallium-based alloys enables rapid mechanical replenishment of electrodes and dynamic reconstruction of reaction interfaces. In Figure 8a, He et al. designed a novel LM flow battery with a gallium–indium–zinc alloy as the electrode and an alkaline electrolyte system [81]. Through the synergistic effect of alloy components, it suppresses hydrogen evolution and dendrite growth while achieving decoupling of energy and power, providing a feasible solution for high-capacity and fast-charging energy storage scenarios. In a strongly alkaline environment, gallium can form a stable and reversible redox couple, providing fundamental potential support for the battery. The intrinsic properties of LMs circumvent the dendrite issue of solid electrodes and create conditions for establishing a genuine flow configuration. In Figure 8b, Athair et al. conducted a feasibility study on room-temperature gallium-based metal–air batteries, confirming that their polarization performance outperforms traditional zinc-air batteries [169]. The inherent characteristics of LMs allow them to avoid dendrite problems and possess advantages of self-healing and convenient replenishment, laying a foundation for the development of LM–air batteries with flexible configurations. Gallium-based LMs have surmounted the critical limitations of conventional batteries through principle-based innovation, demonstrating remarkable application potential.

### 5.2. Thermoelectric Generator

**Figure 9:**
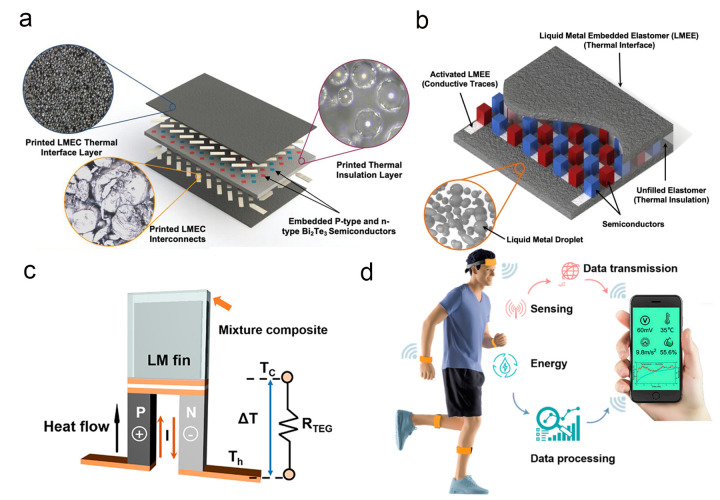
(**a**) Schematic of a stretchable TEG device and micrographs of printed materials consisting of top and bottom thermal interface LM elastomer composites, thermal insulation layer with printed hollow microsphere elastomer composite and encapsulated rigid semiconductors in the middle, and top and bottom LM elastomer composite interconnects [170]. Copyright 2022, Advanced Energy Materials. (**b**) A schematic of the stretchable TEG with a thermally conductive LM embedded elastomer as the material interface on the top and bottom. The LM embedded elastomer contains a mechanically sintered pattern for providing electrical connections between p-type and n-type Bi_2_Te_3_ semiconductors [82]. Copyright 2020, ACS Applied Materials & Interfaces. (**c**) The working principle of LM-enhanced wearable thermoelectric generators [172]. Copyright 2022, Bioengineering. (**d**) Application of LM-WTEG for powering the wearable sensor system [172]. Copyright 2022, Bioengineering.

Traditional elastomer-based TEGs face issues such as insufficient stretchability and low thermal conductivity. LM elastomer composites, formed by combining gallium-based LMs with elastomers, can fulfill dual functions of thermal interface conduction and stretchable interconnection through tailored microstructural design. As shown in Figure 9a, Han et al. constructed a three-layer TEG using 3D printing technology, integrating high-thermal-conductivity thermal interface materials, hollow microsphere-reinforced insulating layers, and stretchable interconnects to achieve automated device fabrication [170]. This design not only enhances the energy conversion efficiency and mechanical stability of the device but also expands the integrated application space of TEGs in flexible scenarios by direct printing on textiles and integrating stretchable heat sinks. The functional integration characteristics of LM embedded elastomers enable the simplification of TEG structures. Their combined thermal conduction and stretchable conductive properties can simultaneously resolve the problems of the encapsulation material thermal insulation and rigid interconnection. In Figure 9b, Zadan et al. integrated Bi_2_Te_3_ semiconductor arrays into LM embedded elastomers, forming conductive traces through mechanical sintering without the need for additional interconnection components, thereby significantly simplifying the manufacturing process [82]. Additionally, the supercooling effect of LM embedded elastomers effectively lowers the freezing temperature of LMs, allowing the device to operate stably in extremely cold environments and broadening its application range.

The flexible application of bulk thermoelectric materials encounters challenges in electrode adaptation and thermal bypass. The low electrical resistance and high stretchability of gallium-based LMs, combined with the flatness of flexible printed circuit boards, can construct efficient composite electrode systems. Lee et al. designed flexible thermoelectric devices adopting a composite electrode structure of “PDMS-encapsulated LMs + flexible printed circuit boards,” paired with flexible brackets to reduce thermal bypass effects [171]. This device not only achieves efficient harvesting of body heat energy but also possesses human-perceivable refrigeration capabilities, enhancing the practical value of flexible thermoelectric equipment through functional integration. The energy conversion efficiency of TEGs is closely related to cold-side thermal management. The latent heat of phase change and high thermal conductivity of gallium-based LMs endow them with notable advantages in heat sink structure design, enabling enhanced heat absorption and release through solid–liquid phase change. Liu et al. developed LM-enhanced wearable thermoelectric generators integrated with flexible finned heat sinks, utilizing the phase change characteristics of LMs to improve heat exchange efficiency while ensuring tight conformity of the device with the skin [172] (see Figure 9c,d). This device successfully self-powers multi-parameter sensors and Bluetooth modules, verifying the feasibility of gallium-based LMs in practical TEG applications.

### 5.3. Energy Harvesting

**Figure 10:**
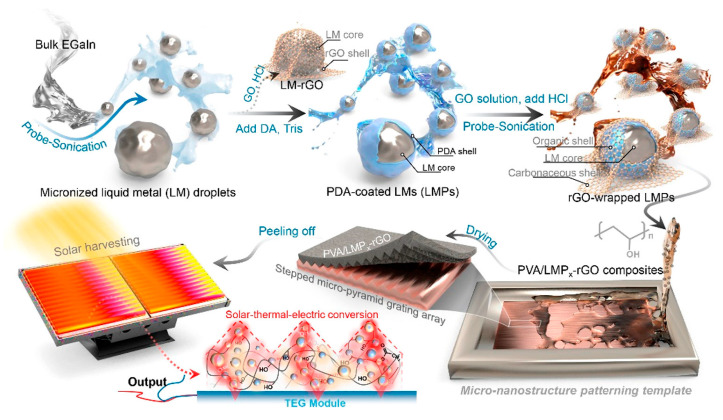
Synthetic roadmap to convert bulk LM to poly vinyl alcohol polydopamine-reduced graphene oxide nanodroplets and the fabrication of poly vinyl alcohol-based photothermal absorber featuring the 3D stepped micropyramid grating array surface [12]. Copyright 2022, ACS Nano.

Gallium-based LMs have emerged as a versatile and high-performance medium for unified energy harvesting. In Figure 10, Yang et al. proposed a dual-interfacial LM nanodroplet structure combined with micro-nanostructure engineering [12]. Using LM as the core, polydopamine as the thermal-insulating interlayer and reduced graphene oxide as the light-absorbing shell, they constructed flexible poly vinyl alcohol-based photothermal absorbers with 3D stepped micropyramid grating arrays for light trapping. This design achieved strong broadband light absorption and high photothermal conversion efficiency, and was further applied to develop a solar-driven TEG with impressive output performance under one sun irradiation, providing a novel material and structural strategy for efficient solar energy harvesting. For waste-heat recovery as another important form of energy harvesting, Dai et al. constructed a thermoelectric generator system using LMs as the heat collection and transfer medium, integrated with commercial thermoelectric modules and an electromagnetic pump [173]. Their experimental prototype achieved considerable open-circuit voltage and energy conversion efficiency under a large temperature difference, which directly verified the engineering feasibility of LM-based TEG systems for waste-heat recovery. Subsequently, Liu et al. applied gallium-based LMs to the thermal charging and discharging processes on both the hot and cold sides of TEGs, and developed rotating permanent magnet electromagnetic pumping technology to realize stable LM circulation at high temperatures [174]. They established a three-dimensional coupled multi-parameter simulation model to reveal the heat transfer and power generation mechanisms, confirming that LM thermal resistance plays a dominant role in the temperature difference in TEG modules. Compared with traditional heat transfer fluids, LMs significantly enhance the power generation performance of waste-heat recovery systems.

## 6. Catalytic

The gallium-based LM catalytic system, leveraging its characteristics of surface dynamic reversibility, self-cleaning anti-coking properties, and tunable electronic structure, offers innovative solutions to the two core challenges of selectivity control and stability enhancement in catalytic science [98]. In the field of electrochemical carbon dioxide reduction, the surfaces of liquid gallium and its alloys exhibit weak adsorption of solid products, effectively addressing the issue of active site blockage caused by carbon deposition or product adhesion in traditional catalysts, thereby achieving highly selective conversion of carbon dioxide to formate or solid carbon at room temperature with significantly improved reaction stability. In thermal catalytic applications, the dynamically flowing surface of gallium-based LMs fundamentally suppresses carbon deposition, and their self-healing properties continuously expose new active sites, enabling reactions such as propane dehydrogenation and methane cracking to maintain long-term stable operation under mild conditions, thus overcoming the bottlenecks of high-temperature sintering and irreversible deactivation in solid catalysts. In the field of single-atom catalysis, gallium-based alloys can serve as liquid electron donors, precisely modulating the electronic structure of single atom sites through the electron spillover effect to construct a dynamic catalytic microenvironment, enabling controllable switching of hydrogenation reaction pathways and overcoming the limitations of static coordination environments and restricted selectivity in traditional single-atom catalysts. The dynamic surface and tailorable electronic structure of LMs provide a distinctive platform for the development of highly efficient and stable catalytic systems [15,16,17,175,176]. This section focuses on three research directions: catalytic carbon dioxide (CO_2_) conversion, high-efficiency thermocatalytic processes, and the burgeoning single-atom catalytic systems.

### 6.1. Catalytic Conversion of Carbon Dioxide

**Figure 11:**
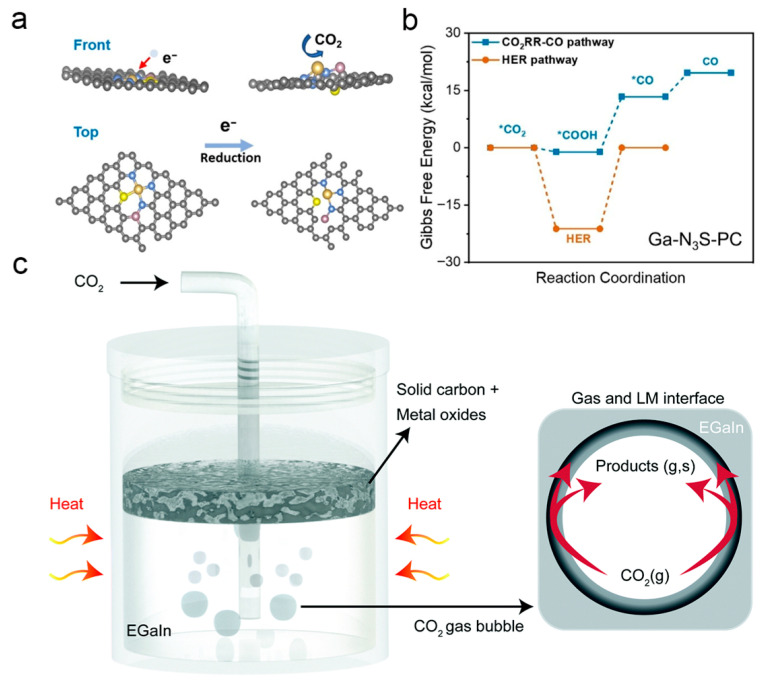
(**a**) CO_2_RR process in Ga-N_3_S-PC catalyst [85]. Copyright 2024, Angew Chem Int Ed Engl. (**b**) Free energy diagrams for CO_2_ reduction to the CO and HER process. Asterisks (*) denote an adsorbed species on the catalyst surface [85]. Copyright 2024, Angew Chem Int Ed Engl. (**c**) Overview of the CO_2_ dissociation process over liquid EGaIn. Representation of the interaction between gaseous CO_2_ and the molten metal for the production of solid carbon and the ensuing generation of Ga oxide is shown on the right [83]. Copyright 2022, Energy & Environmental Science.

Fluxional behavior is essential for catalytic systems to adapt to complex reaction processes, yet the rigid structures of traditional Ga-based catalysts hinder the dynamic regulation of active sites. Zhang et al. constructed a Ga-based catalytic system with a coordination environment of P and S atoms via a spatial confinement strategy, tuning the active centers of the main group element Ga to the atomic scale and endowing it with fluxional behavior starkly different from that of conventional bulk gallium catalysts [85]. In Figure 11a,b, this catalytic system exhibits outstanding performance in the electrochemical CO_2_ reduction reaction (CO_2_RR), achieving a maximum Faradaic efficiency of ~92% for CO production at a potential of 0.3 V vs. RHE. Theoretical simulations confirm that the adaptive dynamic transition of Ga atoms can dynamically optimize the adsorption energy of the *COOH intermediate and refresh active sites in real time, laying a solid foundation for the design and development of dynamic interfacial catalytic systems. Cu-based catalysts possess strong affinity and reactivity towards CO_2_, but their large-scale application is limited by issues such as broad product distribution, sintering and agglomeration during prolonged reactions, and coking-induced deactivation. The dynamic surfaces of Ga-based LMs are rich in active sites, possessing both high surface energy and excellent electronic transfer capability, which can complement the catalytic properties of Cu. Chen et al. developed a Cu-Ga-based LM composite catalytic system, combining the efficient electronic transfer capability of Ga-based LMs with the specific interaction of Cu with CO_2_ to achieve continuous conversion of CO_2_ to solid carbon under ambient temperature and pressure [177]. Using dimethylformamide as the solvent, a solid carbon yield of 2598.7 μmol/h was obtained at 40 °C, and the CO_2_ conversion efficiency reached up to 96% by adjusting the mass ratio of GaIn-Cu (II) salts. This system exhibits favorable stability and recyclability, effectively circumventing the problem of coking deactivation and opening a low-cost pathway for the preparation of high-value solid carbon materials from CO_2_ via non-noble metal catalysis.

The high thermodynamic stability of CO_2_ molecules requires extremely high temperatures for decomposition. Traditional thermal catalytic technologies also rely on solid catalysts and auxiliary reducing agents, resulting in not only high energy consumption but also easy catalytic deactivation due to coking. The EGaIn LM alloy features a low melting point, and its liquid-phase structure can afford a dynamic interface. Additionally, carbon has low solubility and high buoyancy in EGaIn, facilitating product separation. In Figure 11c, Zuraiqi et al. developed a direct CO_2_ conversion method without the need for auxiliary reducing agents. This system achieves a carbon yield of 319 μmol/h at 200 °C and can even realize CO_2_ activation and carbon formation at room temperature [83]. In situ X-ray photoelectron spectroscopy and density functional theory calculations confirm that the final reaction products are solid carbon and gallium oxide, with the catalyst showing no signs of coking-induced deactivation. This approach significantly reduces the energy consumption and process complexity of CO_2_ conversion, providing a new pathway for emission reduction in carbon-intensive industries.

### 6.2. Thermocatalytic

**Figure 12:**
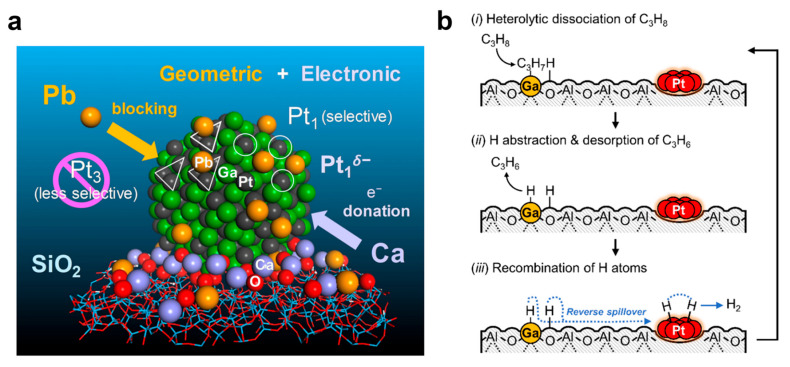
(**a**) Catalyst design concept of the double decoration of the nanoparticulate intermetallic PtGa supported on SiO_2_. The synergy of the geometric and electronic effects is expected to greatly enhance propylene selectivity and the stability of the catalyst [84]. Copyright 2021, Angew Chem Int Ed Engl. (**b**) “Bifunctional Mechanism of Pt-Promoted Ga_x_Al_2-x_O_3 _Catalysts for PDH [180].” Copyright 2025, J Am. Chem. Soc.

In propane dehydrogenation (PDH) reactions, the high-temperature deactivation of Pt-based catalysts stems from side reactions and coke formation, and the synergistic regulation of geometric and electronic effects is pivotal for enhancing stability. As shown in Figure 12a, Nakaya et al. proposed a dual modification strategy for PtGa intermetallic compounds, using Pb to block Pt_3_ active sites for inhibiting side reactions and Ca to construct electron-enriched Pt_1_ sites [84]. Without altering the bulk structure of PtGa, this design enables the long-term stable operation of the catalyst. Traditional methane-to-hydrogen processes exhibit high carbon emissions, and methane pyrolysis, as a low-carbon pathway, requires addressing the balance between reaction efficiency and catalyst stability. LMs possess both heat transfer and catalytic properties, providing a new reaction system for this process. Perez et al. constructed a bubble column reactor using molten gallium as the heat transfer medium and catalyst, achieving efficient methane conversion [178]. The optimized GaOx-Ir-K/Al_2_O_3_ catalyst realized the synergistic improvement of activity and selectivity under high propane concentrations through rational regulation of H_2_ co-feeding amount.

In hydrocarbon dehydrogenation reactions, the influence of hydrogen partial pressure on catalytic activity is closely related to the reaction mechanism. Conventional viewpoints hold that the competitive adsorption of hydrogen inhibits the reaction, yet certain catalysts display a positive dependence. Through in situ characterization and theoretical calculations, Sun et al. confirmed that this characteristic of gallium-based oxide catalysts originates from the mediating effect of metastable gallium hydride [179]. The gallium hydride species formed under H_2_ co-feeding can promote C-H bond activation and inhibit deep dehydrogenation. Even at a low loading of 100 ppm Pt and 1 wt% Ga, the comprehensive performance of this catalyst surpasses that of the benchmark PtSn/γ-Al_2_O_3_ catalyst with 7000 ppm Pt, significantly reducing the consumption of precious metals. The design of PDH catalysts needs to balance high activity and low precious metal consumption, and the bifunctional catalytic mechanism is expected to achieve this goal through the synergistic effect of different active sites. Lee et al. developed a Pt-promoted gallia–alumina composite oxide catalyst, where Ga sites are responsible for C-H bond dissociation and Pt catalyzes H atom recombination as depicted in Figure 12b. They determined the optimal matching ratio by quantifying the functions of active sites, and introduced Ce^3+^ to suppress Pt sintering [180]. This catalyst achieves performance superior to the benchmark catalyst at extremely low precious metal loadings, remarkably improving the utilization efficiency of precious metals. Gallium-based LMs and their derived catalytic systems have made significant progress in thermal catalytic reactions such as PDH and methane pyrolysis through strategies including structural modification and mechanism innovation. Design concepts such as the synergy of geometric and electronic effects, the construction of bifunctional mechanisms, and the mediation of metastable species have effectively addressed the deactivation and selectivity issues of traditional catalysts.

### 6.3. Single-Atom Catalysis

**Figure 13:**
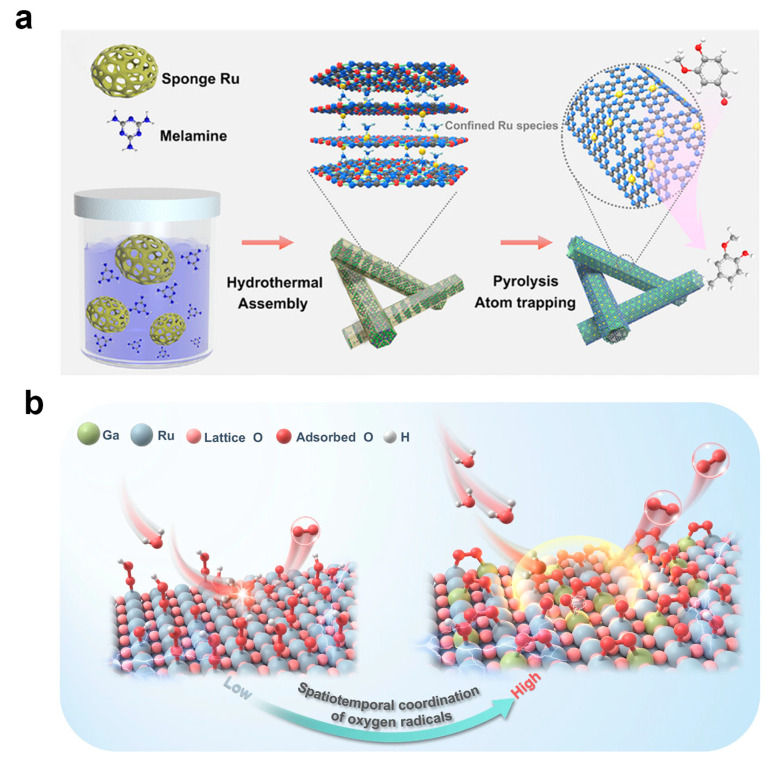
(**a**) Synthesis and characterization of hollow Ru1@m-tube. a) Schematic of the preparation strategy for hollow Ru1@m-tube [181]. Copyright 2025, Angew. Chem. Int. Ed. Engl. (**b**) Schematic illustration AEM pathway of iRuO_2_ and oxide path mechanism pathway of iGa_0.2_Ru_0.8_O_2_ [182]. Copyright 2025, Nat Commun.

Electron transfer stands as one of the fundamental mechanisms governing the selectivity of single-atom catalysts, as the electron density of active sites directly governs the binding strength and conversion pathways of reaction intermediates. A critical challenge in single-atom catalysis lies in precisely modulating the electronic environment of active sites to achieve selective control over complex reaction pathways, an objective that remains difficult to accomplish with conventional catalyst supports. Gallium-based eutectic alloys possess intrinsic characteristics of high electron density and liquid structure, serving as highly efficient electron donors to regulate the electronic environment of single-atom active sites via the interfacial electron spillover effect. As shown in Figure 13a, Song et al. developed a carbon nitride-supported ruthenium single-atom catalyst and constructed a composite system by coating it with a gallium–indium–tin eutectic alloy [181]. Leveraging the directed electron transfer from the alloy to ruthenium single-atom sites, they optimized the desorption kinetics of target products and achieved selective modulation of hydrogenation and hydrodeoxygenation reactions.

For the oxygen evolution reaction, conventional catalytic pathways are often constrained by high energy barriers, creating a need for alternative mechanisms that can circumvent these limitations through innovative catalyst design. The oxide path mechanism circumvents the energy barriers of traditional pathways through direct oxygen radical coupling, with its fundamental mechanism lying in the realization of spatiotemporal coordination of oxygen radicals. Gallium single atoms exhibit the ability to stabilize oxygen radicals and form dynamically tunable chemical bonds, acting as specific radical donors to induce synergistic effects with adjacent active sites. As illustrated in Figure 13b, Wang et al. incorporated gallium single atoms into a ruthenium oxide nanocrystal framework, and through the precise coupling between oxygen radicals stabilized at gallium sites and those at ruthenium sites, they successfully established an oxide path mechanism dominant catalytic pathway [182].

## 7. Summary and Outlook

Gallium-based LMs have achieved leapfrog development from basic research to application transformation in four cutting-edge fields including sensors, biomedicine, energy and catalysis, relying on their excellent electrical conductivity, high thermal conductivity, low toxicity, deformability and dynamic interface properties. They demonstrate irreplaceable technical advantages and market potential. In the sensor field, their high sensitivity with a maximum of 66.07 MPa^−1^, wide detection range reaching 660% strain, self-healing ability and multifunctional integration are particularly prominent. Not only have core devices such as ultra-sensitive electronic skin and flexible strain sensors been developed, but also industrial breakthroughs have been made. Yunnan LM Valley has successfully supplied thermal management modules for Huawei servers, reflecting strong market demand. In the biomedicine field, gallium-based LMs are centered on good biocompatibility, with the maximum tolerated dose of EGaIn nanoparticles as high as 700 mg kg^−1^. Key progress has been made in intelligent drug delivery, minimally invasive medical devices, biomedical imaging contrast agents and nerve repair. Multiple technologies have entered the preclinical research stage, providing a new path for the innovation of precision medicine and minimally invasive surgery. In the energy field, EGaIn-MnO_2_ stretchable batteries can cycle stably over 100 times under 100% strain with an areal specific capacity of 3.8 mAh cm^−2^. The composite material of EGaIn and paraffin achieves a photothermal conversion efficiency of 83.3%. LM batteries and thermoelectric conversion systems show potential for large-scale application in flexible energy storage and distributed energy respectively. In the catalysis field, the methanol oxidation activity of liquid platinum–gallium catalysts is three orders of magnitude higher than that of traditional solid platinum catalysts. They exhibit ultra-high activity and selectivity in electrochemical CO_2_ reduction with an initial potential as low as −310 mV, methanol-to-hydrocarbon conversion with catalyst lifetime increased by 14 times, propane dehydrogenation and other reactions, offering new ideas for green catalysis and energy conversion. All four fields present common characteristics of inter-disciplinary integration and rapid transformation from basic research to application, with gradually improved technical maturity and accelerated industrialization process.

Currently, gallium-based LM technology still faces multiple challenges. At the material level, it is necessary to address issues such as oxidation control, long-term stability and cost reduction. The scarcity and price volatility of gallium resources also exert certain pressure on industrialization. At the technical level, the sensor field has problems of packaging reliability and signal crosstalk. The biomedicine field needs to improve long-term safety evaluation and regulatory approval processes. The energy field confronts bottlenecks in high-temperature operation and sealing technology. The catalysis field must overcome engineering problems such as LM leakage and product separation. At the industrialization level, there is a lack of unified material standards, testing methods and quality control systems. However, policy support, emerging industrial demands, advancements in related technologies and inter-disciplinary integration provide broad opportunities. In the long run, sensors will develop in-depth towards intelligence, multifunctional integration and wearability, and smart clothing and electronic skin are expected to achieve large-scale commercialization. The biomedicine field will accelerate clinical translation, with precision medicine and intelligent medical devices becoming core directions. The energy field will achieve breakthroughs in flexible energy storage technology, and LM batteries are expected to occupy an important position in the grid-scale energy storage market. The catalysis field will focus on greenization and industrialization, realizing commercial application in CO_2_ conversion, fine chemicals and other fields. By strengthening basic research, improving standard systems, promoting industry–university–research integration and talent training, gallium-based LM technology is expected to break through existing bottlenecks and become a core material support for promoting technological upgrading and sustainable development in related fields.

## Data Availability

No new data were created or analyzed in this study.

## Abbreviations

The following abbreviations are used in this manuscript:

LM	Liquid metal
EGaIn	Eutectic gallium–indium
EGCG	Epigallocatechin gallate
Hap	Hyaroxgapatite
TEG	Thermoelectric generator
CO_2_	Carbon dioxide
PDH	Propane dehydrogenation
